# Artificial intelligence software to detect small hepatic lesions on hepatobiliary-phase images using multiscale sampling

**DOI:** 10.1007/s11604-025-01859-6

**Published:** 2025-08-29

**Authors:** Shogo Maeda, Yuko Nakamura, Toru Higaki, Ayu Karasudani, Tatsuya Yamaguchi, Masaki Ishihara, Takayuki Baba, Shota Kondo, Dara Fonseca, Kazuo Awai

**Affiliations:** 1 Diagnostic Radiology, Hiroshima University, 1-2-3 Kasumi, Minami-ku, Hiroshima City, Hiroshima 734-8551 Japan; 2 Fujitsu Research, Fujitsu Limited, 4-1-1 Kamikodanaka, Nakaharsa-ku, Kawasaki City, Kanagawa 211-8588 Japan; 3 Hiroshima Prefectural Hospital Organization, 10-52 Motomachi, Naka-ku, Hiroshima, 731-0011, Japan

**Keywords:** Artificial intelligence, Multiscale sampling method, Detectability, Hepatobiliary-phase images

## Abstract

**Purpose:**

To investigate the effect of multiscale sampling artificial intelligence (msAI) software adapted to small hepatic lesions on the diagnostic performance of readers interpreting gadoxetic acid-enhanced hepatobiliary-phase (HBP) images.

**Methods:**

HBP images of 30 patients harboring 186 hepatic lesions were included. Three board-certified radiologists, 9 radiology residents, and 2 general physicians interpreted HBP image data sets twice, once with and once without the msAI software at 2-week intervals. Jackknife free-response receiver-operating characteristic analysis was performed to calculate the figure of merit (FOM) for detecting hepatic lesions. The negative consultation ratio (NCR), percentage of correct diagnoses turning into incorrect by the AI software, was calculated. We defined readers whose NCR was lower than 10% as those correctly diagnosed the false findings presented by the software.

**Results:**

The msAI software significantly improved the lesion localization fraction (LLF) for all readers (0.74 vs 0.82, *p* < 0.01); the FOM did not (0.76 vs 0.78, *p* = 0.45). In lesion-size-based subgroup analysis, the LLF (0.40 vs 0.53, *p* < 0.01) improved significantly with the AI software even for lesions smaller than 6 mm, whereas the FOM (0.63 vs 0.66, *p* = 0.51) showed no significant difference. Among 10 readers with an NCR lower than 10%, not only the LLF but also the FOM were significantly better with the software (LLF 0.77 vs 0.82, FOM 0.79 vs 0.84, both *p* < 0.01).

**Conclusion:**

The detectability of small hepatic lesions on HBP images was improved with msAI software especially when its results were properly evaluated.

## Introduction

Metastatics are more common than primary hepatic tumors such as hepatocellular carcinoma [1, 2]. The mortality rate of patients with hepatic metastasis from colorectal cancer is lower and their prognosis is better after surgical resection [3]. Therefore, the improved management of patients with hepatic metastasis requires the accurate detection of even small lesions.

Gadoxetic acid (Gd-EOB-DTPA: EOB)-enhanced hepatobiliary-phase (HBP) MRI is highly sensitive for the detection of hepatic lesions, because the contrast agent is hepatocyte-specific [4]. In an earlier meta-analysis examining the detection sensitivity for liver metastasis from gastrointestinal cancer, sensitivity and specificity were 93% and 95%, respectively [5]. A later meta-analysis of liver metastasis from colorectal cancer found that sensitivity and specificity were higher with EOB-MRI than contrast-enhanced CT (93% and 87%, respectively, vs 82% and 74%, respectively [4]. Consequently, EOB-MRI has been strongly recommended for diagnosing liver metastasis especially in the setting of preoperative evaluation [6, 7]. However, small lesions, especially those near hepatic vessels, may be missed on EOB-MRI scans, because both hepatic lesions and vessels are hypo-intense on HBP images [8, 9].

The use of Artificial intelligence (AI) in radiology has been on the rise and AI software for the detection of lesions on chest radiographs and lung CT scans has been developed [10, 11]. Although the detection by readers of lesions on HBP- and lung CT images is similar, the development of AI software for lesion detection on HBP images is complicated. First, a three-dimensional (3D) approach is required to differentiate between hepatic vessels and lesions and more computer resources are needed to compare the validity of the 3D- with the conventional 2D approach [12]. Also, there are too few types of datasets to which the 3D approach can be applied [13]. The lesion size variety is greater with respect to hepatic- than lung lesions and AI software to detect variously-sized hepatic lesions is required [14].

The lightweight detection architecture adapted to small lesions using multiscale sampling (ms) method is a 2D approach incorporated in deep learning-based AI software; it can detect hepatic lesions of various sizes on HBP images [14]. We hypothesized that this msAI software can assist in the detection of even small hepatic lesions on HBP images. Therefore, we investigated whether it improved the diagnostic performance of readers tasked with detecting hepatic lesions on HBP images.

## Materials and methods

This retrospective, observational study was performed in accordance with relevant guidelines and regulations of our institution. This report adheres to the checklist for AI in medical imaging guidelines [15].

### Lightweight detection architecture adapted to small lesions using multiscale sampling method

Details have been reported [14]. Briefly, this model was developed using HBP images from 45 cases (31 for training and 14 for validation), all of which were completely independent from the cases included in the observer performance study, thereby eliminating the risk of data leakage. The image acquisition parameters (including scanner model, sequence type, and imaging protocol) used for model development were identical to those used in the observer study, as all images were acquired at the same institution under the same protocol. Therefore, there is no domain shift between the model development data and the test data.

This model applies multiscale sampling (ms) and a 2D patch using the minimum-intensity projection of 3 orthogonal planes.

- 1. Multiscale sampling
  Depending on the target to be detect, the size of the patches to be sampled for training the AI network should be optimized. Small lesions cannot be detected when the network is trained on large patches; large lesions cannot be detected when the patch size is small. Therefore, the msAI software samples 6-, 12-, and 24-mm patch images.
- 2. 2D patches using the minimum-intensity projection of 3 orthogonal planes
  The detection of lesions on HBP images requires 3D information. Therefore, the patch images were converted from 3 to 2D using the minimum-intensity projection of axial, coronal, and sagittal planes to preserve the 3D information even on 2D images. The binary classification (hepatic lesion yes/no) is obtained using the lightweight 2D deep convolutional neural network model with 2D minimum-intensity projection images of the 3 orthogonal planes as the inputs.

### Study design

This retrospective and observational study was approved by our institutional review board; prior informed consent from participants was waived. Patient records and information were anonymized and de-identified prior to analysis. This study was conducted to evaluate the ability of msAI software to assist in the detection of hepatic lesions on HBP images. Readers with different levels of experience were enrolled, and their performance with and without msAI software was compared in a setting similar to the clinical practice.

### Study population

**Fig. 1:**
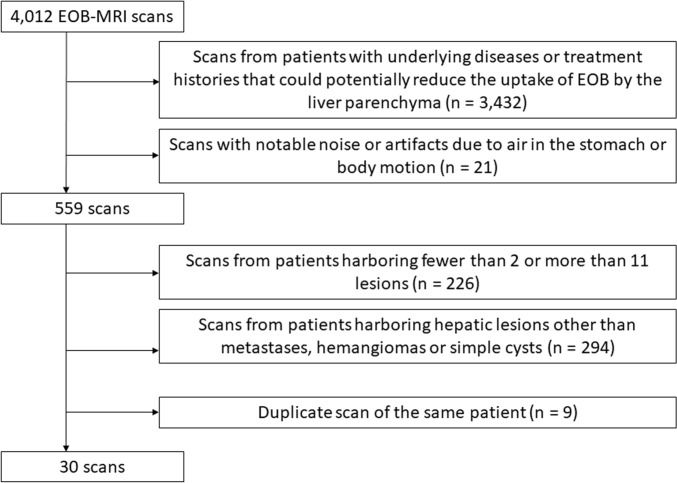
Flowchart of case enrollment

We collected 4,012 scans from patients who had undergone EOB-MRI studies between March 2018 and November 2023. The inclusion criteria included the presence of hepatic metastases, hemangiomas, or simple cysts. The following scans were excluded: (1) scans from patients with underlying diseases or treatment histories, such as chemotherapy and transcatheter arterial chemo-embolization that could potentially reduce the uptake of EOB by the liver parenchyma; (2) scans with notable noise or artifacts due to air in the stomach or body motion; (3) scans from patients harboring fewer than 2 or more than 11 lesions (because a small number of lesions renders their detection too easy, while too many lesions increases the burden on the readers); (4) scans from patients harboring hepatic lesions other than metastases, hemangiomas or simple cysts (other hepatic tumors, calcifications, postoperative scars, or undiagnosed lesions); (5) duplicate scans of the same patient. Finally, HBP images of 30 scans with 186 hepatic lesions were included in the observer performance study (Fig. 1).

The ground truth (GT) for the hepatic lesions was determined by 2 board-certified radiologists (SM and YN with 6 and 21 years of experience, respectively). They were excluded from the observer performance study with reference to all other sequences on EOB-MRI- and PET scans obtained with fluorine 18 fluorodeoxyglucose, and on contrast-enhanced CT images. Pathologic findings obtained at definitive surgery were also considered. Patient and lesion details recorded as the GT are presented in Table 1.

**Table 1:** Hepatic lesions

Characteristics	Value
No. of patients	30
Median age years (range)	63 (36–79)
Men/women	17/13
Lesion information	
Number of lesions	186
Median number of lesions per patient (range)	6 (3–10)
Lesion type (*n* = 186)	
Hepatic simple cysts	85
Hepatic hemangiomas	66
Hepatic metastasis	35
Median lesion size [mm] (range)	7.9 (4.1–74.5)
Number of lesions by size [mm]	
All	186
≤ 12.0	137
≤ 10.0	125
≤ 8.0	95
≤ 6.0	35

### Image acquisition

Scans were performed on a 3 T MRI instrument (TRILLIUM OVAL; FUJIFILM, Tokyo, Japan) using a 28-channel coil. EOB (25 μmol/kg, Primovist; Bayer Yakuhin, Osaka, Japan) was injected at 2.0 ml/s and flushed with 20 ml of saline using a power injector (Sonic Shot 50; Nemoto Kyorindo, Tokyo, Japan).

HBP imaging was started 20 min after the EOB injection. Images were obtained with a fat-saturated T1-weighted gradient-echo nature of the sequence with parallel imaging (rapid acquisition through a parallel imaging design; RAPID, FUJIFILM Corporation). The scan parameters were slice thickness and interval 3.0 mm, TR/TE 4.0 ms/1.8 ms, flip angle 15°, field of view 36 cm, and matrix 320 × 224. Only the HBP images were used for the observer performance study. Although other sequences including dynamic MRI scans using EOB were obtained for the clinical studies, they were not evaluated.

### Observer performance study

The number of readers required for the observer performance study was calculated using G*Power 3.1.9.7 [16, 17]. In the observer performance study, lesion-level sensitivity, i.e., the lesion localization fraction (LLF), will be calculated. Although LLF is originally derived from binary outcomes, the calculation of the required number of readers should focus on per-reader LLF, which is treated as a continuous variable. A preliminary analysis, conducted using data from the same dataset used in the observer performance study and involving readers who did not participate in the actual observer performance study, yielded an effect size of 1.15 for the difference in per-reader LLF with and without the msAI software. Based on this effect size, a significance level of 5%, and a power of 80%, the required minimum number of readers was calculated to be 9 using the Wilcoxon signed-rank test. Our study involved 14 readers; they were 3 board-certified radiologists with 5–7 years of experience, 9 radiology residents with less than 4 years of experience, and 2 general physicians.

All readers received standardized instructions, were informed of the software operation methods, and all were trained on 12 patients excluded from the observer performance study. They were informed only that images showing cysts, hemangiomas, or hepatic metastases were included. Post-training, each reader interpreted the HBP image data sets twice, once with and once without msAI software in the concurrent reader mode. In each session, they were instructed to detect and annotate all hepatic lesions, and to rate their confidence level for each detected lesion on a scale from 0 to 100. They were also required to complete each interpretation session within 3 min. To minimize any memory bias, we imposed an interval of at least 2 weeks between the sessions.

**Fig. 2:**
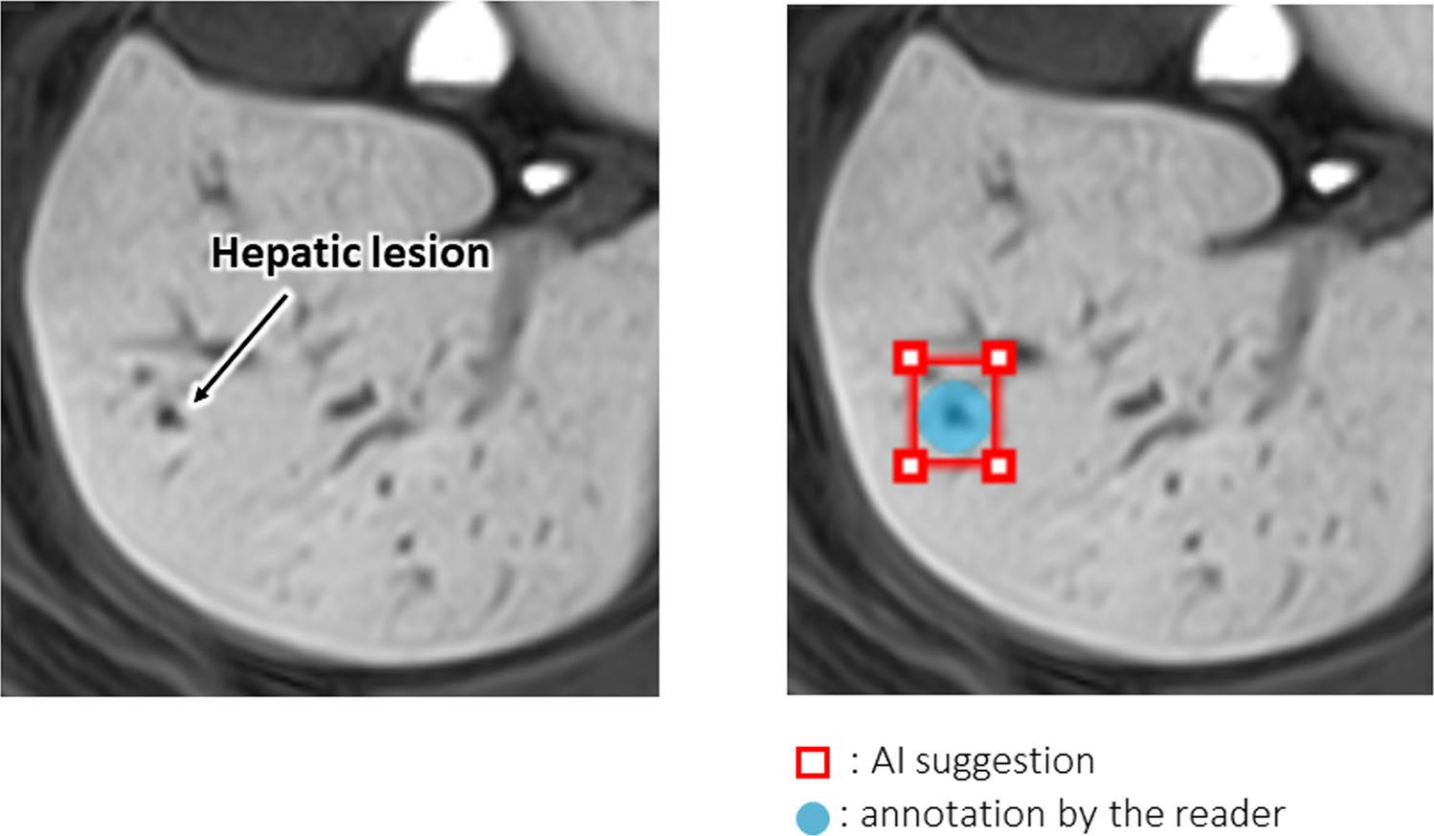
The original graphical user interface program for the observer performance test. The hepatic lesion detected by the reader is circled in blue; a red square shows the lesion identified by the AI software

We created a graphical user interface program for displaying Digital Imaging and Communications in Medicine images and ancillary information, lesion annotations, confidence level inputs, and lesion candidate displays. Lesion annotations recorded by the readers are blue, and suspected lesions identified by the software are red (Fig. 2). The reading environment included one monitor for viewing and inputting ancillary information, one high-resolution monitor for viewing the HBP images (EIZO Radi10 Force RX 240, 21.3 inch, 2 M color), and a personal computer that included the dedicated applications (Intel(R) Xeon(R) W-2104 CPU @ 3.20 GHz, RAM 16 GB).

### Data analysis

Annotations made by the readers were recorded as true positive (TP) when the distance between the center of their annotation markers and the GT were within 3/4 of the diameter of the GT and when the distance was equal or less than 3 mm. As the msAI software evaluates annotations in three-dimensional space, the z-axis was also taken into account. Therefore, annotations placed on adjacent slices were not considered false positives as long as the 3D Euclidean distance between the annotation and the ground truth remained within the defined threshold. The LLF was calculated without using confidence score information. Any detection that correctly localized a lesion—regardless of its confidence score—was counted as a true positive. As a result, LLF was calculated under conditions that inherently allow a relatively high number of false positives. This approach was chosen to comprehensively assess lesion-level sensitivity without discarding low-confidence but correctly localized detections, as the primary goal of this study was to ensure that lesions were detected in the first place. The figure of merit (FOM) for detecting hepatic lesions was calculated using jackknife-free-response receiver-operating characteristic (JAFROC) analysis [18, 19]. Because our evaluation focused on lesion-level detection performance with localization, conventional receiver-operating characteristic (ROC) analysis, which assesses classification performance on a per-case (e.g., per-patient) basis, was not suitable. On the other hand, both free-response receiver-operating characteristic (FROC) and JAFROC allow for multiple lesion markings per image and evaluate localization accuracy. Unlike FROC, which is primarily descriptive and visual (e.g., plotting sensitivity versus false positives), JAFROC enables statistical comparison between conditions (e.g., with vs. without the software) by computing an FOM using a jackknife resampling method.

The negative consultation ratio (NCR), the percentage of correct diagnoses turning into incorrect by the AI software, was calculated [20]. High NCR values indicate that the reader tended to rely too much on the AI software. Negative consultation includes 2 decision patterns; true negative (TN) without and false positive (FP) with the AI software, and TP without-and false negative (FN) with the AI software. The NCR was calculated by dividing the negative consultations by all patterns. TN with and without the AI software are not included in the set of all patterns since the analysis includes only findings that were detected either with or without the software (or both). We labeled readers with an NCR lower than 10% as those who evaluated the results presented by the AI software results correctly.

### Statistical analysis

Statistical analyses were performed using R version 4.5.1 (R Foundation for Statistical Computing, Vienna, Austria). The difference in LLF with and without the msAI software was tested with the paired t test, while the difference in FOM was evaluated using Dorfman–Berbaum–Metz method; a *p* value < 0.05 was considered statistically significant. We also performed subset analysis based on the lesion size using a 6 mm (smallest patch size) threshold and the NCR using a 10% threshold.

## Results

### Standalone performance of the multiscale sampling AI software

The LLF of the AI software for detecting all hepatic lesions was 0.82 and 14 FPs per case. The results of subgroup analysis based on a lesion size of ≤ 12 mm, ≤ 10 mm, ≤ 8 mm, and ≤ 6 mm was 0.77, 0.75, 0.70, and 0.49, respectively (Table 2).

**Table 2:** Lesion localization fraction and FOM for all readers based on the lesion size

	Number of lesions	Without AI	With AI	*P* value	AI (14 FPs/case)
LLF					
All size	186	0.74	0.82	< 0.01	0.82
≤ 12.0 mm	137	0.67	0.77	< 0.01	0.77
≤ 10.0 mm	125	0.65	0.76	< 0.01	0.75
≤ 8.0 mm	95	0.61	0.72	< 0.01	0.70
≤ 6.0 mm	35	0.40	0.53	< 0.01	0.49
FOM					
All sizes	186	0.76	0.78	0.45	–
≤ 12.0 mm	137	0.73	0.76	0.19	–
≤ 10.0 mm	125	0.72	0.76	0.09	–
≤ 8.0 mm	95	0.71	0.75	0.12	–
≤ 6.0 mm	35	0.63	0.66	0.51	–

*LLF* lesion localization fraction, *FOM* figure of merit, *AI* artificial intelligence, *FP* false-positive

### Observer performance study

**Fig. 3:**
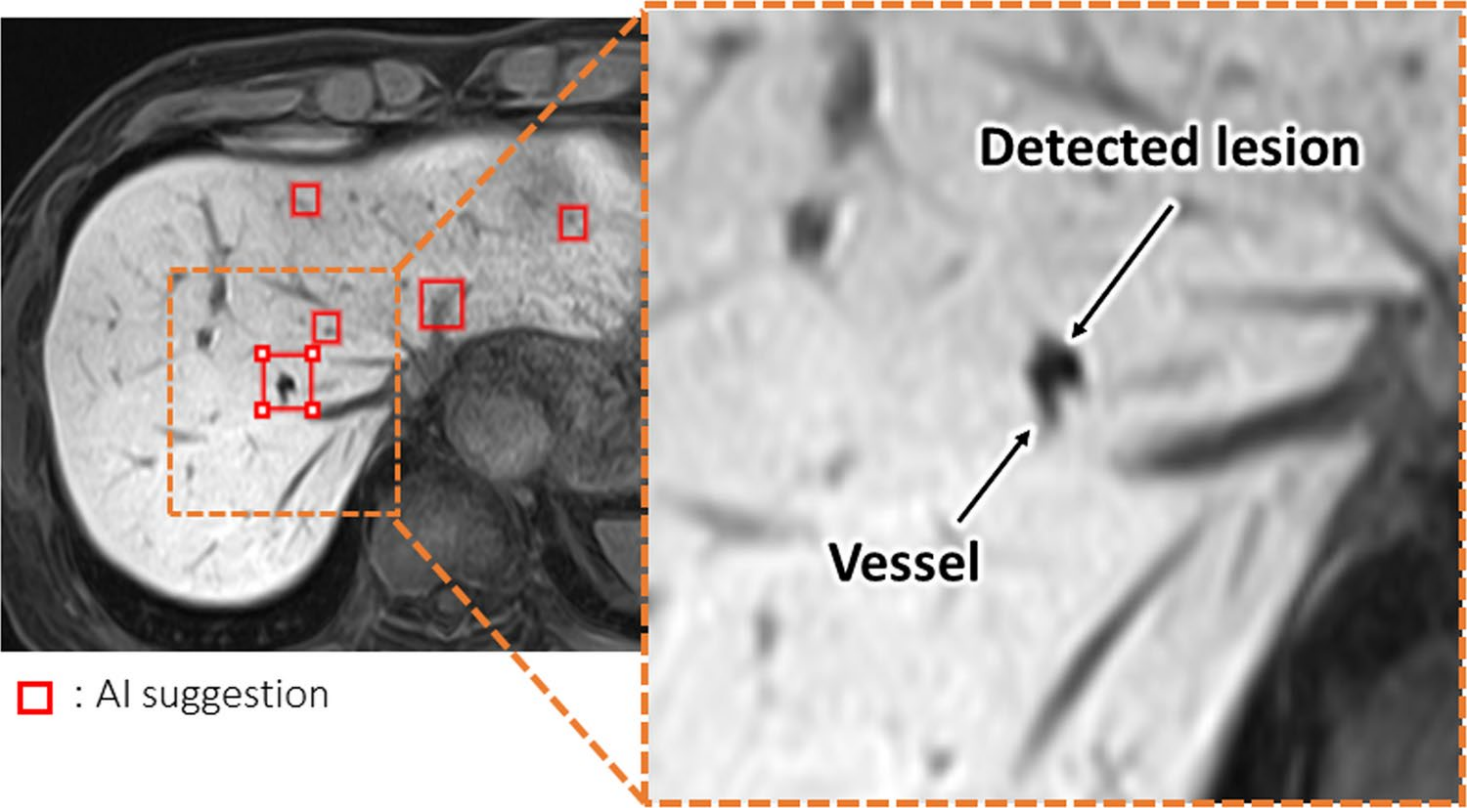
Small hepatic lesion near hepatic vessels. The multiscale sampling AI software detected a small hepatic lesion adjacent to a hepatic vessel (red square). Readers identified this small lesion not identified without the software

The use of the AI software increased LLF significantly for all readers (0.74 vs 0.82, p < 0.01); the FOM tended to increase with the AI software, but not significant (0.76 vs 0.78; *p* = 0.45) (Table 2). According to size-based subgroup analysis of the hepatic lesions, including lesions smaller than 6 mm, LLF was significantly improved by the AI software regardless of lesion size (Table 2, Fig. 3). On the other hand, although FOM tended to improve with the AI software, no statistically significant differences were observed for any lesion size category (Table 2).

**Fig. 4:**
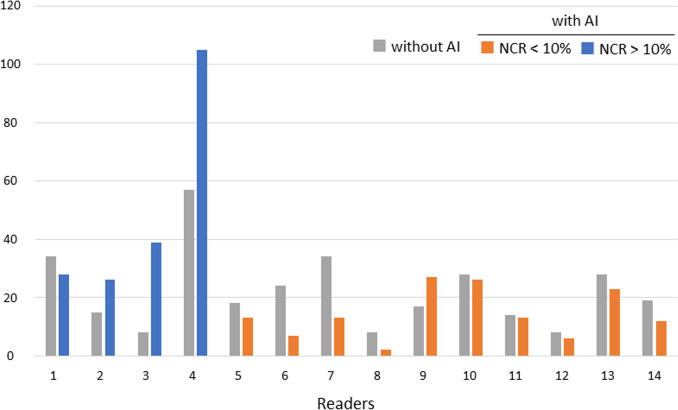
Number of false positives for all 14 readers. *NCR* negative consultation ratio

**Fig. 5:**
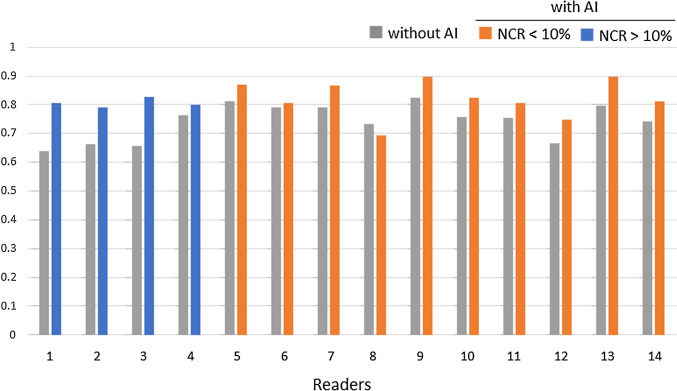
Lesion localization fraction for all 14 readers. *NCR* negative consultation ratio

**Fig. 6:**
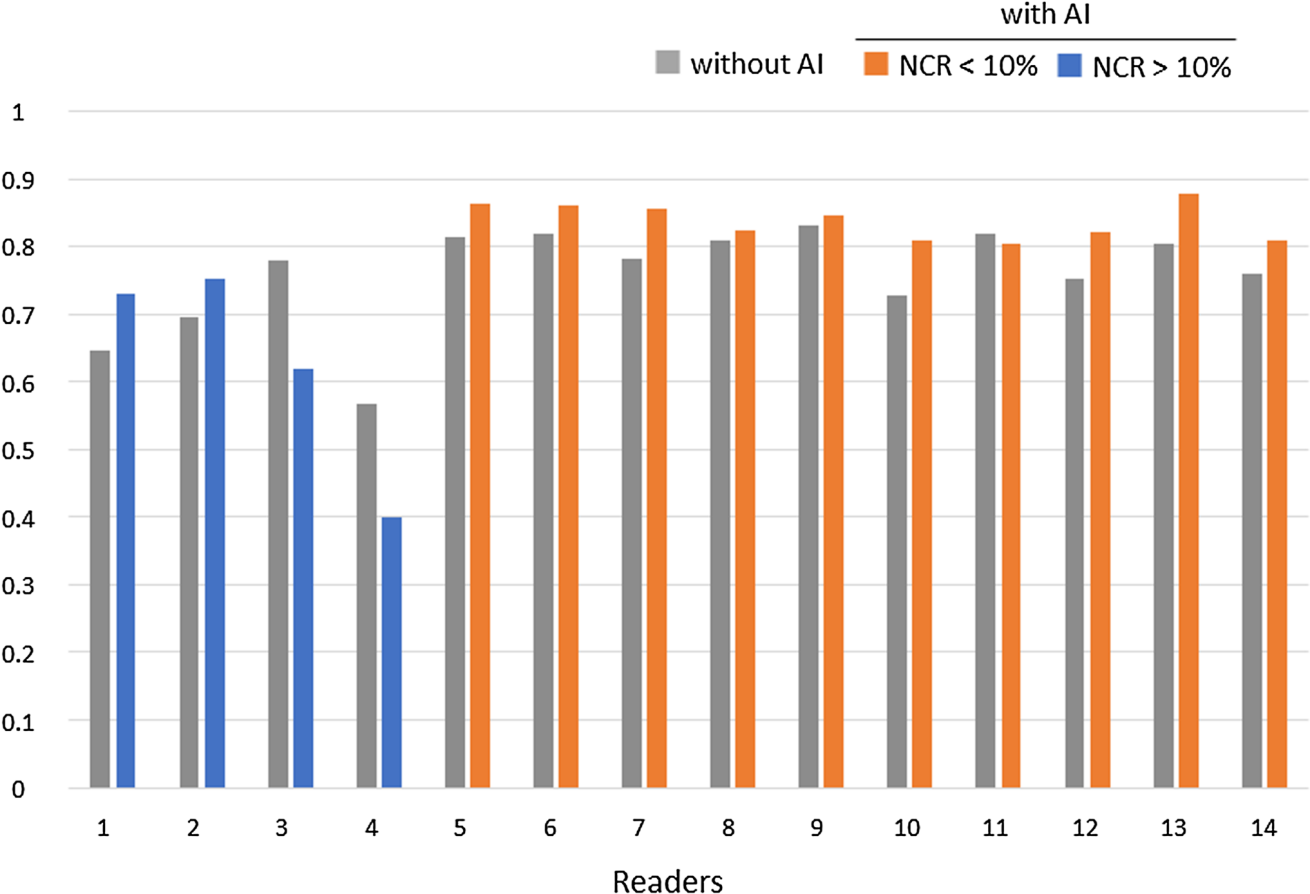
Figure of merit for all 14 readers. *NCR* negative consultation ratio

The NCR was above 10% for 4 readers, including 2 general physicians (readers 1 and 2) and 2 radiology residents with less than 1 year of experience (readers 3 and 4). The other 10 readers with an NCR lower than 10% were radiologists with more than 2 years of experience. In most readers with an NCR higher than 10%, the use of the AI software led to an increase in false positives, whereas false positives decreased in all but one of the readers with an NCR lower than 10% (Fig. 4). In addition, for the 10 readers with an NCR lower than 10%, not only the LLF but also the FOM were significantly higher with the AI software (LLF 0.77 vs 0.82, FOM 0.79 vs 0.84, both *p* < 0.01) (Table 3, Figs. 5 and 6).

**Table 3:** Lesion localization fraction and FOM based on the NCR

	LLF			FOM		
	Without AI	With AI	*P* value	Without AI	With AI	*P* value
All readers (*n* = 14)	0.74	0.82	< 0.01	0.76	0.78	0.45
Readers with NCR < 10% (*n* = 10)	0.77	0.82	< 0.01	0.79	0.84	< 0.01

*LLF* lesion localization fraction, *FOM* figure of merit, *NCR* negative consultation ratio, *AI* artificial intelligence

## Discussion

The LLF, even for lesions smaller than 6 mm, was significantly improved when the msAI software was used. The nodule size that can be detected by the AI software depended on the patch size. Lesion detectability of the AI software using single-scale sampling has been reported to be 0.60 for all lesions at an average of 25 FPs per case. It is limited especially for lesions smaller than 1 cm, because the patch size was 31 mm [21]. The msAI software was designed to detect small lesions in particular; multiple patch sizes, including 6-mm patches, were used and the detectability of hepatic lesions was higher for all lesion sizes and the results were better than when AI software with single-scale sampling was applied [21]. While there are differences in disease targets, imaging modalities, and network architectures between our study and previous report using single-scale sampling [21], the higher LLF observed in our results suggests that the multiscale design may be one of several contributing factors to improved detection performance. These findings indicate that the msAI software has the potential to enhance the detection of small hepatic lesions on HBP images.

**Fig. 7:**
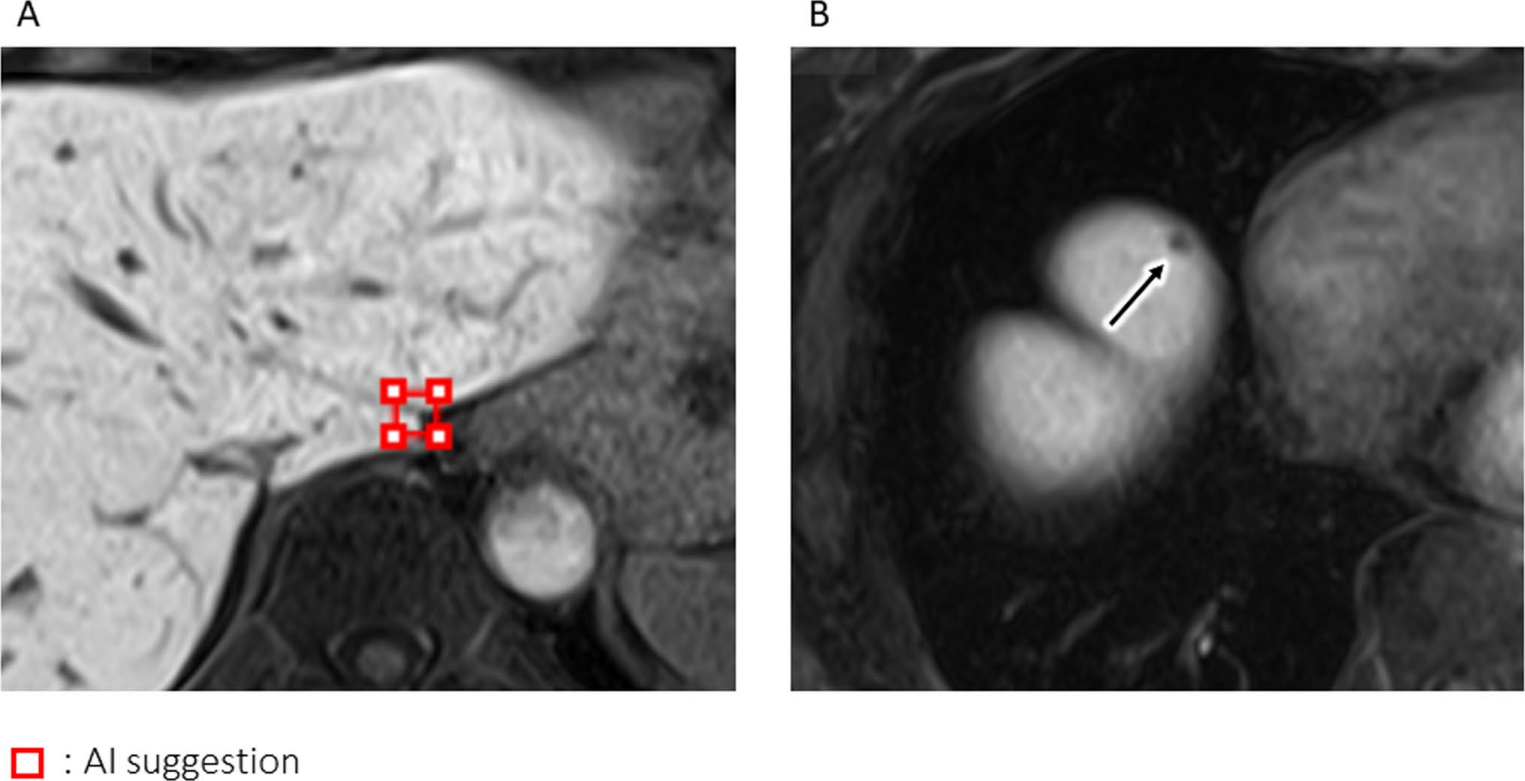
False-positive and false-negative results presented by the AI software. **A** (false positive): No lesion was found at the site noted by the AI software (red square). **B** (false negative): Hepatic lesion near an organ boundary (arrow); it was not detected by the AI software

The AI software tended to increase the FOM with respect to all lesions although the differences were not statistically significant. FP and FN results may be returned by the msAI, particularly when the lesions are in the vicinity of large blood vessels or organ boundaries (Fig. 7). The model used in the msAI software first performs automatic segmentation of the liver region. However, the liver segmentation is not fully accurate, and large blood vessels are not excluded from the segmented region. This limitation can lead to false positives and false negatives, particularly near large vessels or organ boundaries [14]. Consequently, it must be recognized that the software may not improve the FOM and that the software should be improved with including more accurate segmentation in future.

Our study showed that the NCR values tended to be lower for more experienced and higher for less-experienced readers. False positives increased in most readers with an NCR higher than 10% when using the AI software, but decreased in all but one of the readers with an NCR lower than 10%. The FOM was improved significantly only in the 10 readers with an NCR lower than 10%. The automation bias, the tendency to over-rely on automation, can result in too little reader deliberation [20]. As did others [22, 23], we found that less-experienced readers manifested a higher automation bias and tended to interpret the AI results incorrectly. Therefore, attention should be needed using msAI software especially for less-experienced readers.

Although a prolonged reading time improved lesion detection by readers, in the clinical setting, it is limited; the recommended reading time per case is about 15 min [24]. As T1-, T2-, and diffusion-weighted sequences are scanned and dynamic images are yielded at EOB-MRI, the reading time for the HBP is restricted to no more than 3 min. To mimic the clinical situation, our reading time at each interpretation session was limited to within 3 min and our results may reflect the clinical situation.

In our study, the LLF without the AI software was 0.74, which is lower than the sensitivity of 0.93 reported in a previous study [4]. There are several possible explanations for this discrepancy. First, the target lesions were relatively small, with a median size of 7.9 mm in our study. In fact, 137 out of 186 lesions (approximately 74%) were 12 mm or smaller, which may have contributed to the lower sensitivity observed. Second, our analysis was limited to the hepatobiliary phase. It is possible that evaluating additional imaging sequences—such as arterial, portal venous, transitional phases, as well as conventional sequences, including T1-, T2-, and diffusion-weighted imaging—could have improved lesion detection and thereby increased overall sensitivity. These methodological differences should be taken into account when comparing results across studies.

Our study has some limitations. The number of readers was relatively small, although power analysis indicated that its size was sufficient for our objective. Ours was a single-institution retrospective study. As the observer performance test was performed using a single-vendor MR scanner, our findings should be considered to be preliminary and studies involving more readers and scanners from different vendors are needed. Also, we cannot rule out selection bias, because several groups of patients were excluded from this study. For instance, this study excluded patients with hepatocellular carcinoma, cirrhosis, or prior treatments, such as chemotherapy or transcatheter arterial chemo-embolization, which may affect liver parenchymal uptake of EOB. Additional investigations must be performed to evaluate the usefulness of the software in such cases. Finally, although the msAI software used in this study detected all, including benign hepatic lesions, we are developing software that identifies the lesion characteristics (benign vs malignant).

In conclusion, the detectability of small hepatic lesions on HBP images was improved with msAI software especially when its results were properly evaluated.

## Data Availability

The data that support the findings of this study are not openly available due to reasons of sensitivity and are available from the corresponding author upon reasonable request.
